# Geography, seasonality, and host‐associated population structure influence the fecal microbiome of a genetically depauparate Arctic mammal

**DOI:** 10.1002/ece3.5768

**Published:** 2019-11-12

**Authors:** Samantha Bird, Erin Prewer, Susan Kutz, Lisa‐Marie Leclerc, Sibelle T. Vilaça, Christopher J. Kyle

**Affiliations:** ^1^ Forensic Science Program Trent University Peterborough ON Canada; ^2^ Environmental and Life Sciences Graduate Program Trent University Peterborough ON Canada; ^3^ Faculty of Veterinary Medicine University of Calgary Calgary AB Canada; ^4^ Canadian Wildlife Health Cooperative Alberta Node Faculty of Veterinary Medicine University of Calgary Calgary AB Canada; ^5^ Department of Environment Government of Nunavut Kugluktuk NU Canada; ^6^ Biology Department Trent University Peterborough ON Canada

**Keywords:** 16S rRNA, Canadian Arctic, fecal microbiome, muskox, *Ovibos moschatus*, population structure

## Abstract

The Canadian Arctic is an extreme environment with low floral and faunal diversity characterized by major seasonal shifts in temperature, moisture, and daylight. Muskoxen (*Ovibos moschatus*) are one of few large herbivores able to survive this harsh environment. Microbiome research of the gastrointestinal tract may hold clues as to how muskoxen exist in the Arctic, but also how this species may respond to rapid environmental changes. In this study, we investigated the effects of season (spring/summer/winter), year (2007–2016), and host genetic structure on population‐level microbiome variation in muskoxen from the Canadian Arctic. We utilized 16S rRNA gene sequencing to characterize the fecal microbial communities of 78 male muskoxen encompassing two population genetic clusters. These clusters are defined by Arctic Mainland and Island populations, including the following: (a) two mainland sampling locations of the Northwest Territories and Nunavut and (b) four locations of Victoria Island. Between these geographic populations, we found that differences in the microbiome reflected host‐associated genetic cluster with evidence of migration. Within populations, seasonality influenced bacterial diversity with no significant differences between years of sampling. We found evidence of pathogenic bacteria, with significantly higher presence in mainland samples. Our findings demonstrate the effects of seasonality and the role of host population‐level structure in driving fecal microbiome differences in a large Arctic mammal.

## Graphical abstract

We analyzed the microbiome and nuclear microsatellites from muskoxen from two genetically distinct Canadian populations (Mainland and Victoria Island). Our results showed differences in the microbiome reflected host‐associated genetic cluster with evidence of migration between these two regions. We found evidence of pathogenic bacteria, with significantly higher presence in Mainland samples.

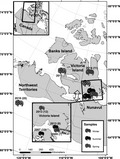

## 1 INTRODUCTION

The gastrointestinal microbiome is a complex microecosystem that plays a significant role in food breakdown and digestion, with profound effects on host health, development, adaptation, and evolution (Bahrndorff, Alemu, Alemneh, & Lund Nielsen, 2016; Hampton‐Marcell, Lopez, & Gilbert, 2017; Kohl, 2017; Zhang et al., 2016). The presence and diversity of different microbial types within microbiomes can be partially explained by spatial and temporal variation between species, populations, and individuals (Bierlich et al., 2018; Gerber, 2014; West et al., 2019). Differences within the microbiome of the same species reflect variable external environmental factors, such as habitat, disease, food availability, and food quality (Amato, 2013; Bahrndorff et al., 2016; Girard, Tromas, Amyot, & Shapiro, 2017). At the population level, local adaptation to the landscape, social structures, and kinship may be reflected in the variable composition of microbiomes (Antwis, Lea, Unwin, & Shultz, 2018; Trosvik et al., 2018; Yuan et al., 2015), while individual microbiomes are impacted by factors such as sex, diet, and health (Aivelo & Norberg, 2018; Bergmann, Craine, Robeson, & Fierer, 2015).

Studying the animal gut microbiome is important toward understanding species ecology and conservation. It provides insight into evolutionary history, and the bacterial community demonstrates phenotypic plasticity that affects the host's capacity to adapt to changing environmental pressures (Antwis et al., 2018; Bahrndorff et al., 2016; Girard et al., 2017). In mammals, the gastrointestinal microbiome has been shown to be affected by captivity (Li et al., 2017), kinship (Yuan et al., 2015), disease (Wasimuddin et al., 2017), and season (Hu et al., 2018). More specifically, many comprehensive microbiome studies have been performed on ungulate species because their unique morphological traits impact the gut bacterial composition, and in return, the bacterial composition impacts fitness (Bergmann et al., 2015; Gruninger, Sensen, McAllister, & Forster, 2014; Koike, Yoshitani, Kobayashi, & Tanaka, 2003; Li et al., 2017; Pope et al., 2012; Qi et al., 2011; Salgado‐Flores, Bockwoldt, Hagen, Pope, & Sundset, 2016). Most ungulates are herbivores that digest cellulose from plant material, including ruminants, which have a chambered stomach and can regurgitate food from their rumen to redigest cud. The ruminant microbiome is dominated by the bacterial phyla *Bacteroidetes* and *Firmicutes* due to their roles in cellulose, carbohydrate, and protein degradation (Gruninger et al., 2014; Li et al., 2017; Pope et al., 2012); for example in sheep, they account for >80% of total ruminal bacteria (Koike et al., 2003). Despite this general similarity among herbivores, the relative abundance of the bacteria making up the microbiome differs between individuals and can be artificially altered by changing factors of diet (Escobar‐Zepeda, De León, & Sanchez‐Flores, 2015; Koike et al., 2003; Tian, Wu, Chen, Yu, & He, 2017). In some populations, diet is altered by shifting seasons and both diet and seasonality have been documented to influence the bacteria community structure in ruminants, including forest musk deer (Hu et al., 2018), and muskoxen (Ungerfeld, Leigh, Forster, & Barboza, 2018). While ungulate and ruminant microbiomes have been generally well‐studied, there is little information on population‐level microbiome variation in these species.

*Ovibos moschatus*, or muskoxen, are ruminants from the same subfamily as sheep and goats (Caprinae), but have a physical appearance that more closely resembles oxen (subfamily Bovinae). They are highly adapted to surviving in arctic conditions with a natural range spanning the Canadian Arctic, from the Northwest Territories and Nunavut into the Arctic islands and western Greenland (Kutz et al., 2017; Reynolds, 1998). Introduced populations occur in Alaska, USA, Yukon and Quebec, Canada, Norway, Sweden and Russia (Kutz et al., 2017). Muskoxen are one of two large herbivores able to survive the arctic environment and one of few large mammals to survive Late Pleistocene mass extinction events approximately 12,000 years ago (Kutz et al., 2015; Raghavan, Themudo, Smith, Zazula, & Campos, 2014). The species has low genetic variation (Prewer, Kutz, Leclerc, & Kyle, 2019; Thulin, Englund, Ericsson, & Spong, 2011) thought to be a matter of populations undergoing several historical and more contemporary bottlenecks that include the following: (a) the Last Glacial Maximum (~18,000 years before present) when populations declined as a result of natural environmental changes in the Beringian ecosystem (Campos et al., 2009); (b) the early 20th century, presumably as a matter of intensive harvest (Anderson, 2016); and (c) most recently, from 2009 onwards, a combination of factors are postulated to explain rapid demographic decline of the two largest natural populations in the world (Banks and Victoria Islands, Canada), including harvest, predation, acute and chronic infectious disease, and other environmental factors (Adamczewski et al., 2016; Anderson, 2016; Cuyler et al., 2019; Kutz et al., 2015, 2017).

Recent climatic changes have led to the Arctic warming two to three times higher than the global annual average (Intergovernmental Panel on Climate Change, 2018). Northern portions of the Arctic are experiencing the most change, with projected rainfall increases to exceed historical averages by over 65% over the course of the twenty‐first century (Bintanja & Selten, 2014) and rain being documented to form ice sheets over accessible foliage (Putkonen et al., 2009), posing obvious threats to muskoxen. With these changing environmental conditions, pathogens (e.g., the nematode species *Umingmakstrongylus pallikuukensis* and *Varestrongylus eleguneniensis*) are dispersing northwards into previously inhospitable environments (Kafle, Lejeune, Verocai, Hoberg, & Kutz, 2015; Kutz et al., 2013). Additionally, some muskox herds have experienced population declines of >50% concurrent with large mortality events associated with the bacterium *Erysipelothrix rhusiopathiae*, a pathogen that had been undetected in the species prior to 2009 (Kutz et al., 2015).

Previous studies have demonstrated that the muskox gastrointestinal microbiome can be affected by season and diet (Andersen‐Ranberg et al., 2018; Thulin et al., 2011; Ungerfeld et al., 2018), though a study by Ungerfeld et al., (2018) found that quality of forage appeared to drive greater changes than season alone in captive muskoxen in Alaska. With known differences in available flora species, length of growing seasons, and pathogen/parasite abundance and diversity between the mainland and Arctic island regions, spatial differences are expected in the microbiomes of muskoxen. It is unclear, however, how the interaction between geographical location and host genotype impacts the muskox microbiome, or that of other wild ungulates for that matter. Relatedness has explained similarities in the microbiome of some species (Yuan et al., 2015), and muskoxen are known to have low genetic diversity with both Norwegian and Greenland populations showing extremely low levels of diversity (Observed Heterozygosity = 0.10 (Thulin et al., 2011)). The lack of genetic variation suggests that relatedness within the species may be high and inbreeding effects are present (Prewer et al., 2019). Despite increased relatedness, the extreme lack of genetic diversity in muskoxen is thought to have a limited impact on their microbiome (Andersen‐Ranberg et al., 2018). To our knowledge, there are currently no studies that investigate both temporal and spatial components of the ungulate microbiome and relate these differences to spatially structured populations, as defined by population genetic parameters; factors that could aid our understanding of how both relatedness and spatial variation influence microbiome diversity.

In the present study, we analyzed the 16S rRNA gene of bacteria from muskox fecal samples from different locations across the Canadian Arctic in an attempt to elucidate the effects of spatial and temporal variation within and between genetically structured populations. These data provide insight into how factors such as genetic diversity, genetic structure, pathogen presence, seasonality, and geographical origin contribute to microbiome dissimilarities in muskoxen and which of these factors act as dominant pressures.

## 2 METHODS

### 2.1 Sample design and collection

**Figure 1:**
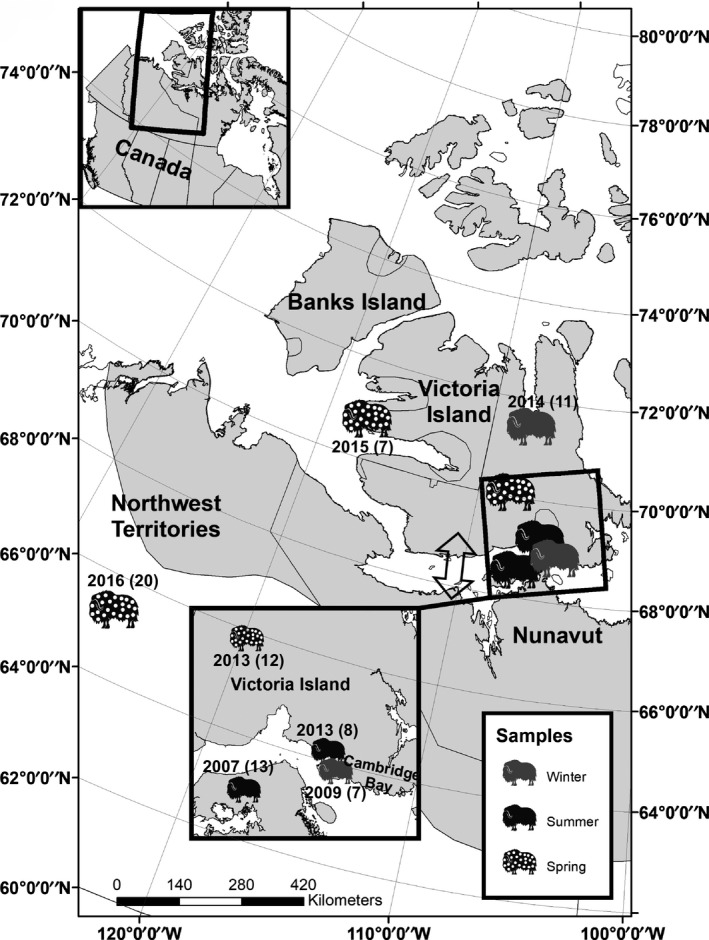
Location, season, and year of collection for sampling sites analyzed in this study. The natural range of muskoxen is shown in light gray. Sample pattern denotes the season of collection (winter, summer, or spring), while year of sampling and sample size, in parenthesis, are indicated by the number next to the marker image. The arrow represents the hypothesized muskox migration route between Mainland and Victoria Island (Kutz et al., 2015)

Fecal samples of wild muskoxen (*n* = 78) were obtained over a 9‐year period from six sampled regions and two genetically defined populations (Prewer et al., 2019), corresponding to mainland portions of the muskoxen range (Kitikmeot Region, Nunavut [*n* = 13 individuals], and Norman Wells, Northwest Territories [*n* = 20 individuals]; hereafter termed “Mainland” population) and Victoria Island (Cambridge Bay, Nunavut [*n* = 15 individuals], Ulukhaktok, Northwest Territories [*n* = 7 individuals], central [*n* = 12 individuals], and northern regions of the island [*n* = 11 individuals]) (Table S1). Samples were collected directly from the rectum of animals harvested by subsistence hunters as part of a community‐based muskox health monitoring program (Tomaselli, 2018) or fresh (<24 hr old) from the ground as part of a study on lungworm range expansion (Kafle et al., 2015). In total, 33 samples were sequenced for the Mainland population (65°N), and 45 samples for the Victoria Island population (70°N). All samples were previously genotyped for individuality using microsatellites loci and sex identification (Prewer et al., 2019). Only samples from genetically unique male individuals were selected to avoid confounding results from female lactation or pregnancy. Samples that were genetically identical to another sample were excluded, even if we knew them to be unique individuals from harvesting. To test for temporal effects, samples were selected between the years of 2007 and 2016 (Figure 1). Samples collected post‐2009 were assumed to be more heavily impacted by infectious disease and hypothesized to have a different microbial diversity due to mortality events in our sampling areas caused by the bacteria *E. rhusiopathiae* (Kutz et al., 2015). Upon collection, all samples were frozen at ambient temperatures, kept on ice at 4°C during transport, frozen at −20°C, and then transferred to −80°C until DNA extraction.

### 2.2 Microbial DNA analysis

Total DNA was extracted from a 0.25 g subsample taken from one fecal pellet per individual and homogenized with the QIAamp PowerFecal DNA kit (QIAGEN). The V3‐V4 fragment of the 16S rRNA gene was amplified with the primers 341F and 805R following the 16S metagenomic Illumina protocol. Primers were modified by adding 0–3 Ns (Tremblay et al., 2015) to increase library diversity during cluster identification (Krueger, Andrews, & Osborne, 2011). Each sample was amplified three times, and the products were visualized on a 1.5% agarose gel stained with ethidium bromide. PCR blank controls consisting of ultrapure water were processed along with the samples to estimate cross‐contamination. PCR triplicates were pooled, and dual 8 bp index combinations were added by an indexing PCR. The DNA concentration per sample was measured using a Quant‐IT dsDNA PicoGreen kit (ThermoFisher) on a FLUOstar Omega microplate reader (BMG Labtech). Each sample was normalized to an equal concentration and pooled as the final library. The final library was run in an Agilent 2200 TapeStation system using the D1000 kit and sent to the University of Guelph (Guelph, Ontario, Canada) Genomics Facility for sequencing by synthesis on the Illumina MiSeq platform using a v3 kit with 600 cycles.

### 2.3 16S rRNA gene data analysis

Two runs of the same library were sequenced independently to act as a technical replicate and test for differences caused by batch effect. Our data were analyzed using the microbial pipeline, Quantitative Insights into Microbial Ecology 2 (QIIME2) v 2018.6 (Bolyen et al., 2019). Each run was analyzed separately to evaluate replicability. We also analyzed our replicate datasets using the software package, Mothur v 1.40.4 (Kozich, Westcott, Baxter, Highlander, & Schloss, 2013) to demonstrate the effects of using different bioinformatics pipelines on the same data (detailed methods can be found in Appendix S1). Using QIIME2, the sequences were trimmed using the *cutadapt* (Martin, 2011) wrapper and denoised using the *dada2* algorithm (Callahan et al., 2016). The sequences were compared with the Silva 132 reference database to form Amplicon Sequence Variants (ASVs) that are better at reducing error by providing single‐nucleotide resolution as opposed to Operational Taxonomic Units (OTUs) that cluster based on a fixed number of nucleotide differences (Callahan, McMurdie, & Holmes, 2017). The sequences were taxonomically classified via BLAST+ consensus and filtered to remove ASVs taxonomically classified as chloroplast, mitochondria, Archaea, or Eukaryota and any sequences that were unclassified at the order‐level (Knight et al., 2018). The libraries were rarefied to equal sequencing depths of 19,997 and 19,529 reads for run 1 and run 2, respectively. To visualize differences in the relationship between samples within technical replicates, Bray–Curtis dissimilarity matrices were imported into the R package *MASS* (Venables & Ripley, 2013; R Core Team, 2017) to create nonmetric multidimensional scaling (NMDS) plots.

A merged dataset from the two QIIME2 runs (see Results for reasoning) was used to calculate alpha diversity (species richness and diversity within a sample) and beta diversity (diversity between environments) indexes using the phylogenetic *core‐metrics* tool. A nonrarefied dataset was used for estimating the number of shared ASVs between populations and for calculating alpha diversity statistics because it provides a more meaningful estimate of diversity by detecting all differentially abundant species (McMurdie & Holmes, 2013). Alpha diversity indexes were tested for normalcy by the Shapiro–Wilk test, and variables were tested for significance by ANOVA or the nonparametric Kruskal–Wallis rank test in R. A PERMANOVA test was used to compare the effects of different variables using *adonis* from the R package *vegan* (Oksanen et al., 2019), and models were ranked using Akaike's Information Criterion (AIC).

The merged dataset was rarefied to an equal sequencing depth of 39,059 reads per sample and was used for generating principle coordinate analysis (PCoA) plots as metrics to detect bacterial diversity between different population groups (Kohl, 2017; Li et al., 2017). The function *core‐features* was used to calculate the core and common microbiomes, similarly to a study on the microbiome of Antarctic whales that also tested for temporal and spatial variability between individuals (Bierlich et al., 2018). These were defined as the ASVs shared between 95% of samples and between 50% and 94% of samples, respectively. The R package *phyloseq* (Kozich et al., 2013; Li et al., 2017; McMurdie & Holmes, 2013) in combination with the package *ggplot2* (Wickham, 2016) was used to create stacked bar plots for visualizing the bacterial abundance between the individual samples.

### 2.4 Population genetics

In order to explore the relationship between microbiome and population of origin, a total of 12 microsatellite loci were genotyped for all samples (Prewer et al., 2019). A discriminate analysis of principal components (DAPC) (Jombart, Devillard, & Balloux, 2010) implemented in the R package *adegenet* (Jombart & Ahmed, 2011) was used to infer population clusters (*K*) and assign individuals to their population of origin. The optimal number of clusters was predicted using the sequential *K*‐means clustering method, using the Bayesian Information Criterion (BIC) for choosing the best number of *k* clusters from one to ten. We also inferred the number of population clusters using the model‐based STRUCTURE algorithm, using the same parameters as set in Prewer et al. (2019). To assess whether microbiome differences observed between Victoria Island and Mainland populations followed the same pattern as the population structure obtained by microsatellite data, the rarefied ASV count data table was converted to presence–absence binary data and used to create a DAPC plot. To assess whether the microbiome bacterial community distance varied predictably with genetic distance, we used the R package *vegan* (Oksanen et al., 2019) to run a Mantel test between microbial Bray–Curtis distance and genetic Euclidean distance. To infer the relationship between genetics and geographical distance, we also ran two partial Mantel tests with geographical distance and genetic Euclidian distance acting as the covariates.

### 2.5 Pathogen detection

Potentially pathogenic bacteria present in the microbiome were screened using the Virulence Factors (VFDB) database (Chen et al., 2005) after taxonomic classification. Any bacteria originating from potentially pathogenic genera were classified to the species‐level by aligning to 16S rRNA gene sequences from the BLAST database with the highest maximum match (>98% identity). The aligned sequences were then identified as potentially pathogenic bacteria by an extensive literature review (Wasimuddin et al., 2017). We calculated the prevalence of all identified pathogens in Mainland and Victoria Island populations and assessed the significance between the two populations using a Fisher's exact test.

## 3 RESULTS

### 3.1 Sequencing and library analysis

Our library consisted of 16S rRNA gene sequences obtained from the fecal samples of 78 individual muskoxen, sampled across six different locations from two genetically distinct populations. The first run resulted in a total of 5,901,931 16S rRNA gene sequences ranging from 32,316 to 130,851 reads per sample with a mean value of 75,655 ± 16,821. The second run resulted in a total of 4,266,439 16S rRNA gene sequences ranging from 25,027 to 97,019 reads per sample with a mean value of 54,693 ± 11,563 (Table S2). Chimera detection of run one removed 2.3% of the total sequences resulting in a final total of 2,494,061 sequences that were clustered into 8,492 single ASVs. Chimera detection of run two removed 2.3% of the sequences resulting in a final dataset of 2,285,635 sequences with 9,565 ASVs. Blank PCR samples (*n* = 4) had varying numbers of reads, ranging from 0 to 3,095 reads postquality control (Table S2). Although this elevated number of reads is probably due to sample cross‐contamination and/or index jumping, the number of ASVs in the two PCR blanks with reads was significantly smaller (76 and 119 ASVs) when compared to the fecal samples (minimum = 368; average = 744 ± 174, Table S3).

Despite fewer total reads sequenced in the second run, NMDS plots showed a similar clustering pattern between samples for both runs and confirmed the replicability of our experiment (stress = 0.055, where <0.01 is ideal to true dissimilarities and <0.05 is excellent). Furthermore, the samples largely clustered by population of origin (Mainland vs. Victoria Island), while four samples clustered in between the two groups (Figure S1). Similar patterns were observed in the NMDS plot produced for the two individual runs of the dataset analyzed by Mothur, even though the dataset produced contrast results to QIIME2 in terms of number of unique sequences and total OTUs. Findings from a comparison between QIIME2 and Mothur are available in Appendix S1.

We merged the two technical runs to describe the bacterial abundances in the muskox microbiome and estimate diversity indices. The final data library consisted of 9,958 ASVs and was rarefied to an equal sequencing depth of 39,059 reads, ensuring a high Good's coverage index (>99%) per sample. The total number of ASVs after rarefaction was 9,832.

### 3.2 Muskox microbiome

**Figure 2:**
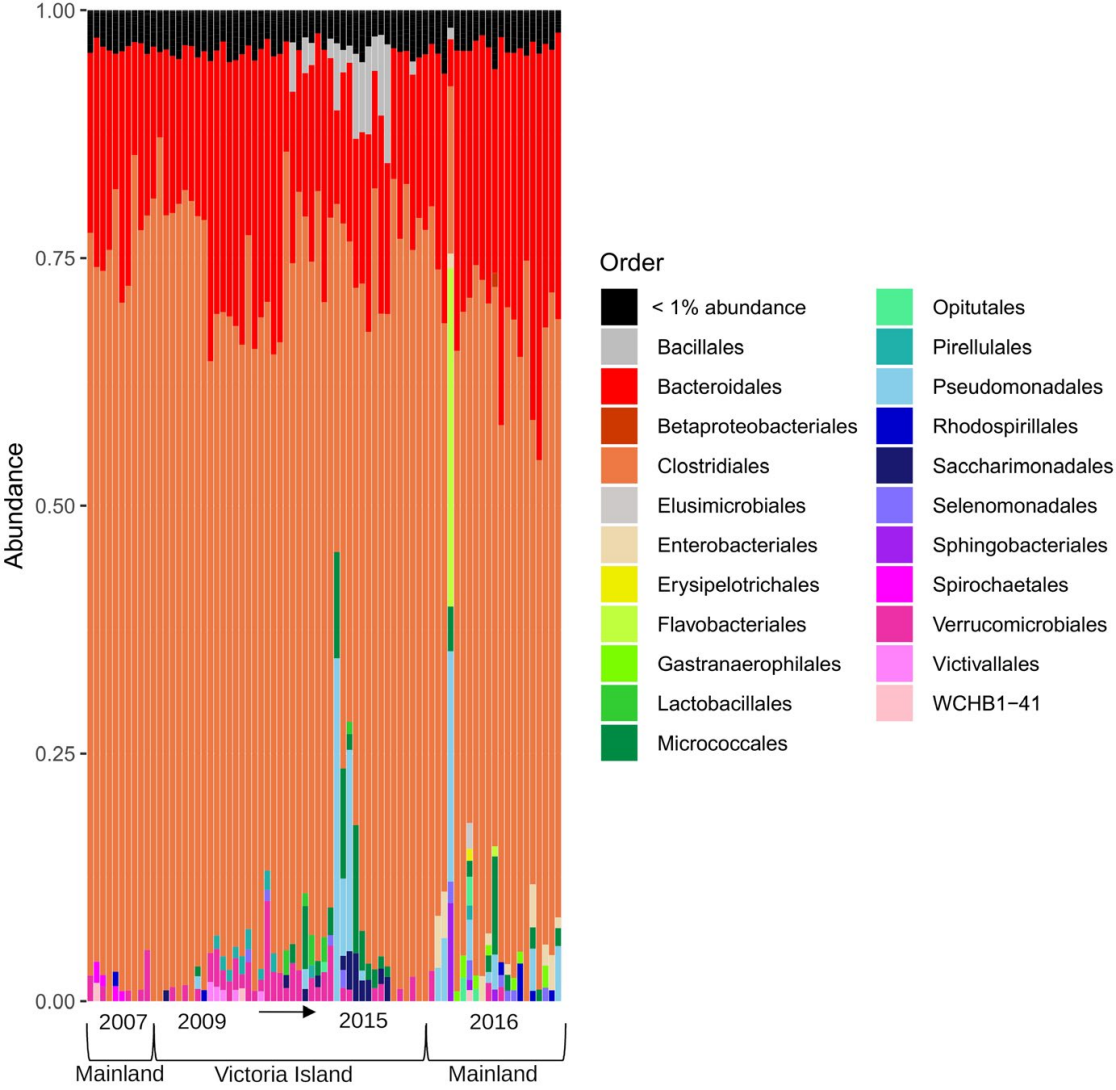
Taxonomic relative abundance plot of the bacterial orders from the merged sequencing run as classified by QIIME2. Samples are organized by year of collection

The fecal microbiome derived from both sequencing runs, merged and processed using QIIME2 and the *dada2* wrapper, was dominated by two main bacterial phyla; *Firmicutes* (83% of the total sequences) and *Bacteroidetes* (7%). Within these phyla, the two most dominant orders were *Clostridiales* (98%) and *Bacteriodales* (89%), respectively (Figure 2). At family level, the *Firmicutes* phyla were composed of *Ruminococcaceae* (66%) and *Lachnospiraceae* (13%). Within the *Ruminococcaceae* family, the most common genera were *Ruminococcaceae* (70%) and *Ruminiclostridum* (4%), while 12% was uncultured. Within the family *Lachnospiraceae*, various *Lachnospiraceae* genera (47%) and *Roseburia* (7%) composed the assemblage, while 11% was uncultured. Within the Bacteroidetes phyla, *Rikenellaceae* (33%), *Bacteroidaceae* (29%), and *Prevotellaceae* (9%) were the dominant families. Within the *Rikenellaceae* family, the *Rikenellaceae RC9 gut group* (47%) and *Alistipes* (44%) were the dominant genera, while for *Bacteroides*, 100% of the total sequences were from the *Bacteroidaceae* family.

The core microbiome (ASVs shared in >95% of fecal samples) of muskoxen was composed of only six shared ASVs: three of which belonging to the bacterial genus *Ruminococcaceae UCG‐005*, and one from each taxon *Bacteroidaceae Bacteroides*, *Lachnospiraceae Roseburia*, and *Clostridiales Family XIII*. Separating the samples by population showed that Victoria Island samples shared a core microbiome of 31 ASVs (48% *Ruminococcaceae*, 19% *Bacteroidaceae*, 16% *Lachnospiraceae*, and 6% *Rikenellaceae*), while Mainland samples shared only three ASVs belonging to the bacterial genus *Ruminococcaceae UCG‐005*. The common microbiome (ASVs shared between 50% and 94% of all samples) consisted of 184 shared ASVs (55% *Ruminococcaceae*, 10% *Lachnospiraceae*, 9% *Bacteroidaceae*, and 7% *Christensenellaceae*).

We detected two samples (308s13134 and 406s2C) with possible environmental contamination. Unlike most of the samples, these were abundant for *Pseudomonadales* bacteria (35% and 23% of total reads, respectively, against 1% average for other samples). One sample from the Mainland population collected during winter (406s2c) showed similar bacterial abundances as Arctic snow, with *Pseudomonadales*, *Flavobacteriales*, and *Sphingobacteriales* as the most abundant orders (Bowman et al., 2012). Grubb's test for outliers identified both samples as significant outliers within their respective populations based on the abundance of *Pseudomonadales* bacteria (*p* < .05), and both samples were excluded from further analyses.

### 3.3 Spatial and temporal differences in muskox fecal bacterial communities

**Figure 3:**
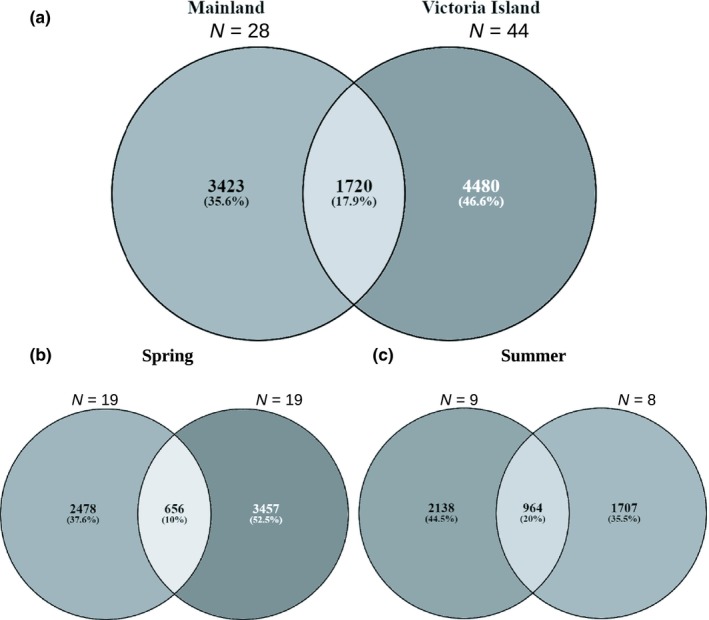
Venn diagrams represent the number of shared ASVs from (a) nonrarefied data between Mainland and Victoria Island populations, and between (b) spring and (c) summer seasons. Putative migrant individuals were removed. Numbers in parenthesis indicate the percentage of total ASVs that were unique or shared between the two populations

**Figure 4:**
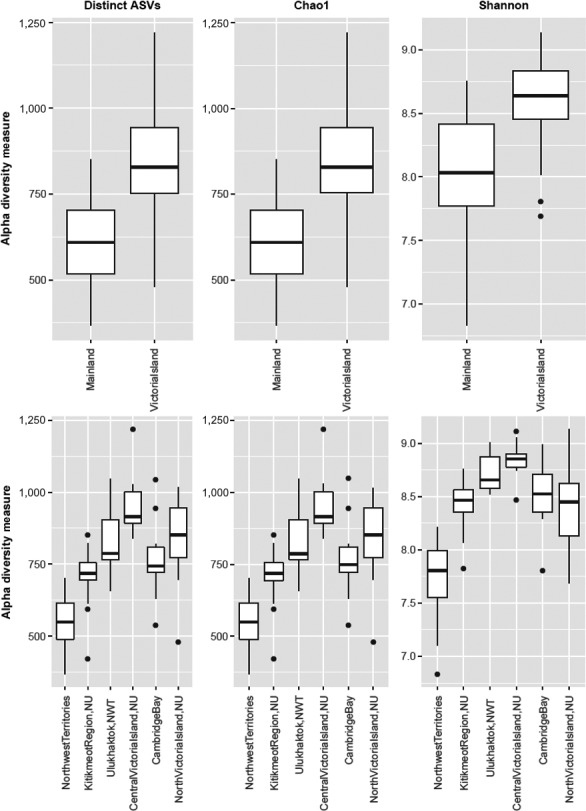
A comparison of alpha diversity metrics of nonrarefied data between Mainland and Victoria Island (top) and between all sampling regions (bottom). Mainland regions: Norman Wells, NWT (denoted Northwest Territories) and Kitikmeot Region, NU. Victoria Island regions: Ulukhaktok, NWT; Central Victoria Island, NU; Cambridge Bay, NU; North Victoria Island, NU

Of the 9,958 ASVs prerarefaction within the muskox fecal microbiome, 1,720 of those were shared between Victoria Island and Mainland populations when putative migrant samples were removed (see “Population structure” subsection for reasoning) (Figure 3). The number of distinct ASVs per sample was highest in Victoria Island, specifically Central Victoria Island (Figure 3b). Alpha diversity statistics Chao1 and Shannon indices were used to assess species richness and diversity between the two populations, respectively (Figure 4). In accordance with the number of distinct ASVs, both species richness and diversity were higher in Victoria Island opposed to Mainland. Chao1 values ranged from 479 to 1,219.75 in Victoria Island and 284 to 851 in Mainland. Shannon's index values were only slightly higher in Victoria Island with values ranging from 5.76 to 9.13 and 5.76 to 8.76 in Mainland (Table S3). Population of origin was shown to affect both the Chao1 richness index (ANOVA, *p* < .05) and Shannon's diversity index (Kruskal–Wallis, *p* < .05).

**Figure 5:**
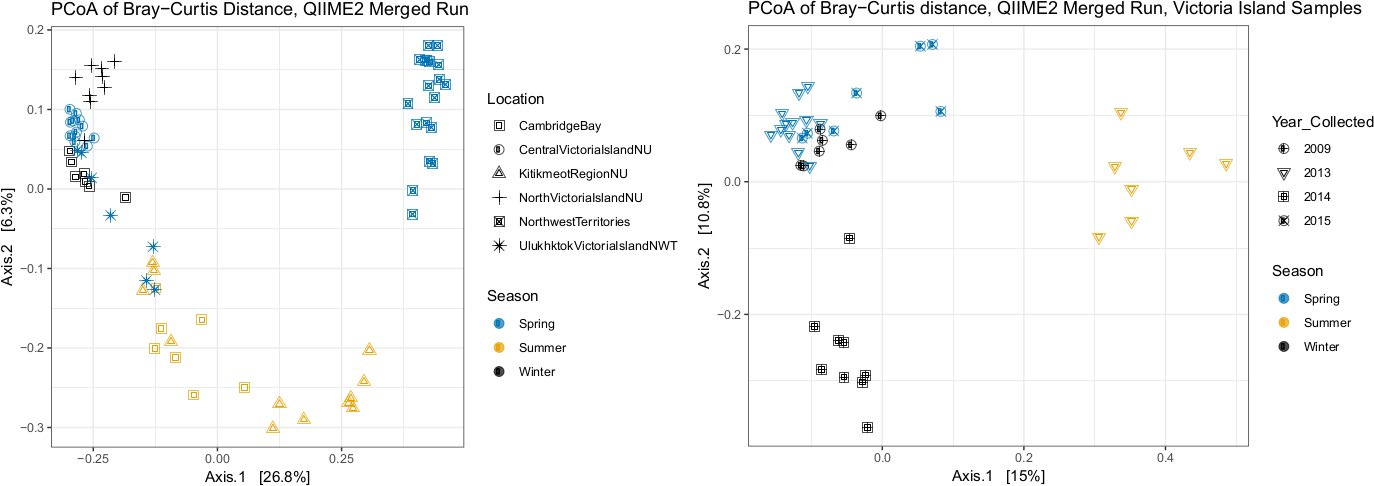
Principal Coordinates Analysis (PCoA) plots of the Bray–Curtis dissimilarity index matrix. Left: Plot for all samples analyzed in this study. Legend shows different sampling locations (represented by different symbols) and season (different colors). Right: Within‐site variation among the Victoria Island samples based on year of sampling (symbols) and season (colors). The percent of variation explained by each axis is shown in square brackets

A PCoA plot of all samples identified a clear separation between the Mainland and Victoria Island populations. Four individuals sampled from Kitikmeot Region, Nunavut, (Mainland) grouped more closely to individuals from Cambridge Bay (Victoria Island) (Figure 5).

In order to test for temporal differences, a PERMANOVA test was used to analyze the effects of each variable of interest using *adonis*. The year of collection was defined as samples collected pre‐2009 and post‐2009 when disease pressures in our sampling area were proposed to be higher. The geographic separation between Mainland and Victoria Island had the greatest effect on the bacterial community (*R* ^2^ = 0.203, *p* = .001), followed by seasonality (*R* ^2^ = 0.128, *p* = .001) and year (*R* ^2^ = 0.034, *p* = .005). Each variable was found to have a significant effect on the bacterial community, so AIC was used to compare and rank the models. When considering all pairwise interactions, the relationship between sampling region and seasonality had the overall strongest influence on the bacterial community (AIC_c_ = 222.543, *w*_i_ = 0.871) while the effect of sampling year had the least (AIC_c_ = 245.508, *w*_i_ = 8.982 × 10^−6^). No models, with the exception of region when variables were assessed individually (AIC_c_ = 230.959, *w*_i_ = 0.988), were able to surpass a 95% confidence set (Symonds & Moussalli, 2011).

### 3.4 Interpopulation seasonal differences in muskox fecal bacterial communities

To test the effect of seasonality on the muskox microbiome, we analyzed samples from Victoria Island collected during spring (*n* = 19 individuals), summer (*n* = 8 individuals), and winter (*n* = 17 individuals). Seasonality is shown to affect the microbial community in abundance (ANOVA, *p* = .014), richness (Kruskal–Wallis, *p* = .017), and diversity (Kruskal–Wallis, *p* = .001). Minor separation can be seen in the PCoA analysis of Victoria Island samples (Figure 5). There were more *Cyanobacteria* in the spring season than both summer and winter months (mean 16% increase, *p* < .001), while the abundance of *Firmicutes* (*p* = .147), *Proteobacteria* (*p* = .616), and *Tenericutes* (*p* = .757) bacteria stayed relatively similar across all seasons. Together, *Firmicutes* and *Bacteriodetes* represented over 90% of the total sequences across all seasons, with a mean increase of 28% in *Bacteriodetes* in summer months (*p* = .001).

### 3.5 Population structure

**Figure 6:**
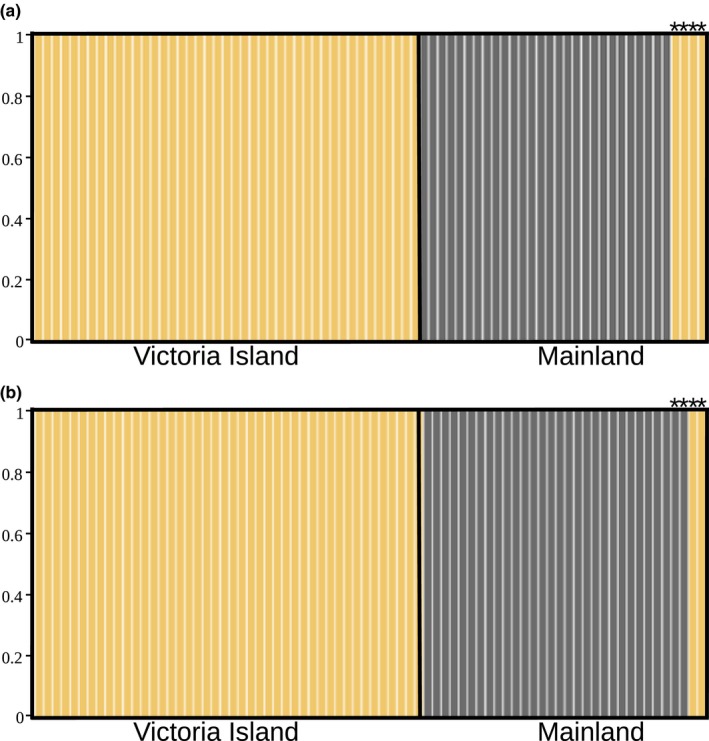
DAPC plots showing the number of clusters (*K* = 2) for fecal microbiome (a) and nuclear microsatellites (b). Each column represents a different individual, and colors represent the assigned population to each sample. Samples were grouped by population of origin (Victoria Island or Mainland). Putative migrant samples are identified by asterisks shown above the bars

DAPC and STRUCTURE assignment tests based on microsatellite data, and the DAPC based rarefied ASV count table both recovered two population clusters (*K* = 2, Figure S2) that were spatially segregated (Victoria Island vs. Mainland, Figure 5). There was a strong positive correlation between the host microbial community distance and the genetic distance (Mantel test, *r* = 0.64, *p* = .001), even when accounting for spatial variation (partial Mantel test, *r* = 0.46, *p* = .001). Likewise, there was a positive correlation between the host microbial community distance and geographical distance when controlling for genetic distance (partial Mantel test, *r* = 0.59, *p* = .001). Similar to the PCoA (Figure 5), four samples collected from Mainland (Kitikmeot Region, Nunavut), grouped with samples collected from Victoria Island (Figure 6a) based on ASV presence. Considering microsatellite genotypes (Figure 6b), two of these four individuals, 101s6C and 101s6E, clustered with Victoria Island samples using both DAPC and STRUCTURE (Figure S3) assignment methods. These four putative migrant samples were removed when identifying the number of unique ASVs in each population to provide the most conservative number of shared ASVs between Mainland and Victoria Island populations.

### 3.6 Potential pathogen diversity in muskox fecal bacterial communities

A total of nine putative pathogenic genera were detected by QIIME2, including bacteria from the order *Rickettsiales* and the genera *Streptococcus*,* Bacilli*,* Yersinia*,* Mycoplasmataceae*, *Arthrobacter*,* Pseudomonas*, and *Erysipelothrix*. Within the nine putative pathogenic genera detected, 47 ASVs were identified. Five of the 47 identified ASVs were classified at species‐level by QIIME2: four ASVs from the genus *Pseudomonas* (*P. baetica*,* P. syringae*,* P. thivervalensis*, and *P. viridiflava*), and one from the genus *Clostridium* (*C. orbiscindens*). Of these, *P. baetica* and *P. syringae* are known plant pathogens while the other three identified ASVs were nonpathogenic bacteria, as per a literature review. The remaining 42 ASVs were unclassified at species‐level by QIIME2, and thus, a representative sequence from each was aligned to 16S rRNA gene sequences from the BLAST database at the highest maximum match (Table 1). A literature review of the resulting alignments resulted in 33 ASVs being classified as nonpathogenic and nine known pathogens being identified, including *Arthrobacter koreensis/A. luteolus* (100% BLAST identity match),* Enterococcus casseliflavus* (100%),* Escherichia fergusonii* (100%), *Serratia fonticola* (100%), *Streptococcus gallolyticus* (100%), *Mycoplasma* sp.* clone A33* (98%), *P. baetica* (100%),* P. fluorescens* (100%), and *P. syringae* (100%). Among the shared ASVs, four out of eight pathogenic bacterial genera differed significantly in their abundance between Mainland and Victoria Island (*p* < .05), with all showing higher abundance in Mainland.

**Table 1:** Potential pathogens and their respective prevalence (%) in Mainland and Victoria island populations

Genus and corresponding OTU IDs	BLAST	Query Cover %	Ident %	Likely pathogenic?	Prevalence %		# Samples Mainland (Season & Year)	# Samples Victoria Island (Season & Year)
					Mainland (*n* = 32)	Victoria Island (*n* = 43)		
Arthrobacter; uncultured	*Arthrobacter koreensis*/*Arthrobacter luteolus* (KY660477.1)	100	100	Yes; Arthrobacter luteolus has prev. been isolated from human wounds^1^	31%	40%	10 (Spring, 2016)	1 (Winter, 2009) 1 (May, 2013) 5 (Summer, 2013) 10 (Winter, 2014)
Enterrococcus; uncultured	*Enterococcus casseliflavus* (MH413022.1, MH376403.1)	100	100	Yes; prev. isolated from humans as the cause for bacteremia infections—considered alone to have low pathogenic potential^2^	0%	2%	–	1 (Summer, 2013)
Streptococcus; uncultured	*Streptococcus gallolyticus* subsp. *pasteurianus* (MG547251.1, MH493716.1)	100	100	Yes; associated with infection in humans^3,4^	3%	0%	1 (Spring, 2016)	–
Yersinia; uncultured	*Serratia fonticola* strain Polovragi/S2 (MH282401.1)	100	100	Yes; a human pathogen associated with abscess following trauma prev. isolated from environmental samples^5^	3%	0%	1 (Spring, 2016)	–
Mycoplasma; ambiguous taxa	Uncultured *Mycoplasma* sp. clone A33 (MH245146.1)	100	98	Yes; prev. isolated from blood of bats and may be associated with hemotropic mycoplasma^6^	19%*	0%	1 (Summer, 2007) 5 (Spring, 2016)	‐
Escherichia‐Shigella; unknown	*Escherichia fergusonii* (MH635312.1)	100	100	Yes, associated with infections of human wounds and diseases in animals^7^	66%*	33%	2 (Summer, 2007) 19 (Spring, 2016)	1 (Winter, 2009) 2 (Winter, 2014) 3 (Summer, 2013) 8 (Spring, 2013)
Pseudomonas; ambiguous taxa	*Pseudomonas fluorescens* (MH620734.1)	100	100	Yes; an opportunistic pathogen associated with bacteremia in humans^8^	38%*	0%	12 (Spring, 2016)	–
	*Pseudomonas syringae* pv. maculicola (LC012019.1), *Pseudomonas syringae* pv. actinidiae (MH101370.1)	100	100	Yes; a plant pathogen^9^				
Pseudomonas; uncultured	*Pseudomonas baetica* strain FE24 (MH620733.1)	100	100	Yes; an opportunistic fish pathogen^10^	25%*	0%	8 (Spring, 2016)	–

Note

ASVs with a significant higher prevalence in the Mainland population are marked with an asterisk (*Fisher's exact test, *p* < .05).

We detected the presence of an ASV from the genus *Erysipelothrix*, which is a bacterium of particular interest as it has been associated with mass die‐offs across Victoria and Banks Islands. However, the sequence isolated from our library was identified as an uncultured environmental sample, aligning to the reference *E. rhusiopathiae* genome at only 93% identity (GenBank accession number: AP012027). Another bacterium, *Yersinia pseudotuberculosis*, has also been responsible for deaths in Canadian muskoxen (Blake, McLean, & Gunn, 1991). The bacterial sequence we found from the genus *Yersinia* was most alike *Serratia fonticola* with a 100% identity match. However, when we aligned the sequence using BLAST to *Y. pseudotuberculosis*, the identity match returned 98% suggesting a high similarity to the bacteria that caused the death of 20 muskoxen on Banks Island in 1986 (Blake et al., 1991).

## 4 DISCUSSION

The data presented in this study suggest that variation in the gastrointestinal microbiome of muskoxen in the Canadian Arctic is reflected by factors of geography, seasonality, and host‐associated population structure. The sampled regions divided into two genetically distinct population groups; those being designated the Mainland and the Island populations. We found a higher pathogen prevalence in the Mainland populations, and our data suggest that the geographic distance and environmental differences between these populations categorizes the microbiome into two distinct microbial clusters. Host‐associated genetic clusters between these populations impacted the bacterial community despite spatial variation and provided some evidence of migration between the two groups. Within population, seasonality impacted microbial diversity with no significant differences between years of sampling.

Contextualizing temporal and spatial variation in the muskox microbiome may be advantageous to allow such data to be used as a screening tool for population management based on diet preference, disease, and the protection of habitats in a changing environment (Bahrndorff et al., 2016). With increasing temperatures and newly expanding pathogens in the Arctic, muskoxen are directly affected by changes in the environment and may be indirectly affected by the change in species abundance or composition of their microbiomes. The microbiome provides a secondary level of immune protection, but can also increase susceptibility to illness potentially threatening overall fitness and further decreasing population sizes (Li et al., 2017). Muskoxen have extremely low levels of genetic diversity, which may undermine their capacity to locally adapt (Prewer et al., 2019), whereas bacteria have much shorter generation times. For these reasons, it is important to understand and monitor changes in the microbiome of muskoxen to make informed conservation decisions.

### 4.1 Differences in muskox fecal bacteria communities

Similar to other ruminants, the major bacterial groups in the muskox fecal microbiome were *Firmicutes* and *Bacteroidetes* (Bergmann et al., 2015; Gruninger et al., 2014; Sundset, Præsteng, Cann, Mathiesen, & MacKie, 2007). These phyla are common in herbivorous ungulates due to their roles in fiber and carbohydrate degradation, as well as immune system support in the gastrointestinal tract (Fernando et al., 2010). Salgado‐Flores et al., (2016) found that the muskox gastrointestinal tract was dominated by *Firmicutes* and *Bacteriodetes* with lesser amounts of the phyla *Tenericutes* and *Cyanobacteria*. While the two major bacterial phyla were the same, we found our muskox fecal samples to have a low abundance of *Tenericutes* and *Proteobacteria*. *Proteobacteria* includes many pathogenic genera, such as the *Escherichia*, *Pseudomonas*, and *Yersinia* genera found within our samples, but it is also symbiotic to the plants that make up the muskox diet and may be naturally found in the intestinal tract (Lock & Wellehan, 2015). The *Ruminococcaceae* family composed the majority of the sequences from *Firmicutes* phyla, and this bacteria family is known to help with digesting highly lignified plant material and made up the majority of the core and common microbiome of muskoxen (Andersen‐Ranberg et al., 2018; Salgado‐Flores et al., 2016).

The Canadian muskox bacterial communities were found to be similar to previous fecal microbiome studies from muskoxen from Norway and Greenland (*n* = 3 and 39 individuals, respectively) (Andersen‐Ranberg et al., 2018; Salgado‐Flores et al., 2016; Ungerfeld et al., 2018). The core genus *Ruminococcaceae UCG‐005* was also reported in all muskoxen samples from Norway and Greenland, while the other three core genera were present in low abundances and were not shared by all samples (Andersen‐Ranberg et al., 2018). Andersen‐Ranberg et al., (2018) hypothesized the difference in relative bacterial abundances between Eastern Greenland and Norway was due to dietary differences, although the authors did not explicitly test this hypothesis. The diversity indices for muskoxen were congruent with the high gut bacterial diversity of ruminants and conformed to the values seen in Norwegian muskox as well as other ungulates, such as sheep, musk deer, and reindeer (Kittelmann et al., 2013; Li et al., 2017; Salgado‐Flores et al., 2016).

Muskoxen inhabit a harsh environment and require a set of specialized and unique bacteria in their digestive system. They have evolved physical traits such as a larger rumen capacity and slow food passage rates to aid digestion of low‐quality and high‐fibrous plant material characteristic of the Arctic (Ihl & Klein, 2001). These adaptations allow muskox to receive more energy from the cellulose being fermented by bacteria into volatile fatty acids in the rumen (Gruninger et al., 2014; Ihl & Klein, 2001). Fecal samples collected from Victoria Island showed a higher bacterial diversity when compared to Mainland, even when considering separate sampling locations. Although these two populations are not too geographically distant (89.08–1,021.93 km), Mainland populations from the Low Arctic (corresponding to Southern Arctic ecozone) experience higher quality forage throughout the year when compared to the Arctic Archipelago region (or Northern Arctic ecozone) where Victoria Island is located. The Canadian Arctic Archipelago has a polar climate (Bliss et al., 2015), with low annual average temperatures and presence of prostrate shrub tundra vegetation (<5 cm), while the Low Arctic is characterized by the presence of graminoid and erect‐shrub tundra (<40 cm) (Walker et al., 2005). Seasonal changes within the Arctic are characterized by drastic light–dark cycles and a shift in available flora due to the freeze–thaw cycles of top soils (Bliss et al., 2015). We hypothesize that muskoxen from Victoria Island need a wider repertoire of gastrointestinal bacteria throughout the year to be able to digest the highly variable forage between seasons, which may justify the higher bacterial diversity seen in this population.

When seasonality in Victoria Island was considered, species abundance, diversity, and richness fluctuated. We observed a higher bacterial diversity in spring when compared to summer, confirming previous results that described a more rapid digestion of forage in spring due to the gain of rumen bacterial diversity (Barboza, Peltier, & Forster, 2006). This may also be due to an availability of higher quality forage during summer months, suggesting the host may modulate bacterial diversity as it can survive with fewer bacteria compared with winter seasons when forage is limited or in decay condition (Barboza et al., 2006). The PCoA results for Victoria Island also showed that within geographical locations, samples clustered by seasonality. We observed that summer and spring samples clustered together, independently of sampling location within the island. The only exception to this pattern was winter samples that formed two separate clusters, while one cluster was unique in relation to the other seasons, the winter samples from Cambridge Bay clustered with spring samples from Central Victoria Island and Western Victoria Island areas. Muskoxen have the ability to regulate their rumen bacterial diversity based on food quality and abundance. During winter seasons and when food abundance and quality is low, muskoxen do not regulate ruminal conditions (Barboza et al., 2006) and spend more time processing food (Lawler & White, 2003). Food intake during midwinter was observed to be as low as spring (Barboza et al., 2006), which may justify the similarity between these two seasons as it correlates with the bacterial diversity in the rumen.

### 4.2 Microbiome as a population marker

Population of origin was found to affect the muskox microbiome, given a clear separation in the population structure of the microbiome between the Mainland and Victoria Island populations as identified via microsatellite markers of the host. We noticed four muskox microbiome samples collected from the Mainland were more closely related to the fecal bacterial composition of Victoria Island samples. Two of these samples clustered with Victoria Island samples based on both microsatellites and bacterial diversity, while the other two samples clustered with Victoria Island only for microbiome structure and their host microsatellite data clustered with Mainland samples. These results indicate a markedly different genetic and bacterial composition between Mainland and Victoria Island, with a potential movement between Victoria Island and the Mainland. The microbiome of these potential migrants may have not yet acquired the bacterial ASVs characteristic of the Mainland or the microbiome may be reflecting individual kinship. Wild ponies (Antwis et al., 2018) and gopher tortoises (Yuan et al., 2015) have shown to reflect maternal and social structuring in their microbial community, which suggests that genetic lineage may be contributing to individual diversity. The relationship between host genetic lineage and microbiome diversity has been studied in baleen whales, where the individual microbiomes reflected functional similarities to historical terrestrial and carnivorous behaviors (Sanders et al., 2015). Our data suggest that microbiome data may be used to investigate a muskox population of origin and real‐time movement patterns on time scales that may not be feasible with the host population genetic analyses alone. Combined with previous studies that hypothesized muskox migration routes between Mainland and Victoria Island (Kutz et al., 2013), our results suggest muskox migration may be occurring in both northern and southern directions. This evidence is also supported by traditional knowledge and community observation (ML Leclerc, personal communication). However, the microbiome is plastic and can rapidly adapt to environmental and diet changes within a matter of days or weeks (Gerber, 2014). The two samples that only clustered with Victoria Island based on microbiome data may not be showing an adaptation to a new geographical location, but rather showing a distinction from the Mainland microbiome structure due to random differences in plant composition (i.e., diet) and bacterial species present in the environment (e.g., soil, water, and snow).

Previously, population‐level variation of microbiomes had been studied in marine mammals (Bierlich et al., 2018), primates (Degnan et al., 2012; Stumpf et al., 2016), ponies (Antwis et al., 2018), amphibians (Griffiths et al., 2018), reptiles (Yuan et al., 2015), and fishes (Webster, Consuegra, Hitchings, & de Leaniz, 2018). To our knowledge, this is the first study to relate population structure to the microbiome of muskoxen. These types of studies demonstrate the importance of population variation in understanding how microbiomes are important to species survival in context of rapid environmental change. They also provide insight into animal‐environment interactions including population‐level similarity between individuals from the same populations, and effects of stress and external environmental changes in the host microbiome.

### 4.3 Potential pathogens?

A total of nine known pathogens were isolated from the fecal microbiota of muskoxen, including bacteria from the genus *Pseudomonas*, *Arthrobacter*, *Streptococcus*, *Enterococcus*, *Escherichia*, *Serratia/Yersinia*, and *Mycoplasma*. Unlike previously reported for muskoxen from Greenland and Norway, we found fewer genera of pathogenic bacteria in Canada (9 vs. 13). Mainland had a higher proportion of potentially pathogenic bacteria, which might be expected due to the more recent emergence and northern expansion of pathogens to Victoria Island (Kutz et al., 2013). Likely as a matter of our more stringent quality control, only three pathogenic bacterial genera (*Streptococcus*, *Escherichia*, and *Serratia*/*Yersinia)* were found in common with muskoxen from Greenland (Andersen‐Ranberg et al., 2018). Among the potential pathogenic bacteria reported in Greenland, an OTU similar to *Erysipelothrix* was found. Although we also found an ASV classified as *Erysipelothrix*, a stringent quality control showed this ASV was not *E. rhusiopathiae*. We did not have access to the OTU sequence reported in Greenland to confirm the similarity to the ASV from our study and to the *E. rhusiopathiae* reference genome. Our results demonstrate the importance of stringent quality controls when identifying pathogenic bacteria in order to minimize the influence of false‐positive reporting. We acknowledge that despite the best quality controls there are limitations in the confidence of positive results when using the short 16S gene region to identify pathogenic bacteria. This method has been used to identify potential pathogens in other studies involving wild animals, such as cheetahs (Wasimuddin et al., 2017), but we advise future work to include a full‐genome sequencing of the putative pathogens to properly identify different strains.

### 4.4 Effects of sampling and controls

Microbiome studies of wild animals often use nonfresh fecal samples due to their noninvasive nature and inclusivity of the entire digestive tract (Li et al., 2017; Liu et al., 2018; Liu, Zhang, Zhang, Zhu, & Mao, 2016; Pope et al., 2012; Salgado‐Flores et al., 2016). Though contamination is a concern with nonfresh fecal samples, our study corroborates with findings from other muskox microbiome studies that used immediate fecal sampling (Andersen‐Ranberg et al., 2018; Ungerfeld et al., 2018). Sample contamination may also come from integration of bacteria from the extraction kit reagents. Though we did not sequence any extraction blanks to exclusively test for reagent contamination, our negative samples had overall few reads (range = 0–3,095 reads) suggesting any kit contamination would be too low to significantly impact our results (Hornung, Zwittink, & Kuijper, 2019). We also referenced two negative samples with higher read counts against a list of reagent and laboratory contaminants known to microbiome analyses (Salter et al., 2014). One negative sample was free of contaminants, while the other consisted of nine reads from the genera *E. coli*. The remaining samples (*n* = 76) were exactly as expected for muskox fecal microbiome, and our controls allowed us to use a higher sample size to infer population microbial diversity and dynamics (Andersen‐Ranberg et al., 2018; Salgado‐Flores et al., 2016; Salter et al., 2014; Ungerfeld et al., 2018).

The same 16S rRNA gene library was sequenced twice for a technical replicate control and to allow an evaluation of batch effect caused by sequencing. Batch effects (i.e., the inability to have reproducible results across sequencing replicates) have been a growing concern in modern microbial analyses (e.g., Goh, Wang, & Wong, 2017). Despite the lower number of total reads in the second run, we found that the individual runs were comparable and could be merged together for a more comprehensive analysis. Our data suggest that combining technical replicates can increase sequencing depth and provide higher confidence in results by overcoming run‐to‐run variation.

Many bioinformatics pipelines and independent programs exist for microbiome data analyses, such as QIIME (Caporaso et al., 2010), Mothur (Kozich et al., 2013), and USEARCH (Edgar, 2010). Each program makes its own assumptions and varies in performance, computational cost, and ease‐of‐use (Mysara, Njima, Leys, Raes, & Monsieurs, 2017). For a thorough microbiome study, it is important to understand the limitations of each program before beginning data analysis. The results of this study were obtained using the QIIME2 program in combination with the *dada2* wrapper due to this program's ability to handle large datasets and general acceptance within the microbial scientific community.

## 5 CONCLUSION

The bacterial communities present within the gastrointestinal tract of muskoxen are integral to their survival in the arctic environment. Studying internal bacterial communities contributes to our understanding of how this iconic Canadian mammal interacts with its environment in terms of digestion, health, immunity, and adaptation; however, it has been unclear how the microbiome community varies within and between regions on temporal and spatial scales, and further how this variation links to population genetic structure. This study used a combined sequencing run of 16S rRNA gene markers in 78 individuals to explore the differences in abundance and diversity of bacteria within the gastrointestinal tract of muskoxen in an attempt to better understand the drivers of microbiome variation. We found that geographic location was a strong driving pressure to microbial diversity. Within population, seasonality impacted microbial diversity, though time of sampling, described here as changing pressures of disease, showed little effect. Future work should consider sequencing the potential pathogens found in the muskox gastrointestinal tract to provide a greater context of health and host capacity to locally adapt to rapidly changing environments. Secondly, the role that diet plays in altering the bacterial component of the microbiome should be more clearly addressed by sequencing plant DNA using a chloroplast marker. Geographic distance shows to affect community diversity and it should be considered how changing vegetation in different landscapes contributes to bacterial variance in a wild mammal, as well as to elucidate the roles of bacteria in fiber and plant digestion for a large herbivore.

## CONFLICT OF INTEREST

None declared.

## AUTHOR CONTRIBUTIONS

STV and CJK designed the research, SK and LML provided samples, SB, STV, and EP performed the research and analyzed data, SB and STV wrote the paper with input from all authors. All authors edited and approved the final version.

## Data Availability

Raw sequences and ASV tables are available for download from Dryad (https://doi.org/10.5061/dryad.fj6q573q2).
