# Supplementary material for: Geography, seasonality, and host‐associated population structure influence the fecal microbiome of a genetically depauparate Arctic mammal
Source: Ecol Evol. 2019 Nov 12;9(23):13202–17. doi: 10.1002/ece3.5768 (PMC6912892; doi:10.1002/ece3.5768)
Supplement: Supplementary file 1 [file ECE3-9-13202-s001.docx]

Supplementary Text 1

There are many different bioinformatics pipelines available for analyzing microbial datasets, each with their own associated formulas and assumptions. Given the variance in read quality filtering, bacteria clustering methods, and chimera detection (Mysara, Njima, Leys, Raes, & Monsieurs, 2017)⁠, we analyzed our data using two common bioinformatics microbial pipelines: Mothur v 1.40.4 (Kozich, Westcott, Baxter, Highlander, & Schloss, 2013)⁠ and Quantitative Insights into Microbial Ecology 2 (QIIME2) v 2018.6 (Bolyen et al., 2018; Caporaso et al., 2010)⁠. We sequenced two runs of the same library to act as replicates and each replicate was analyzed by each pipeline separately, for a total of four datasets (denoted Mothur Run 1, Mothur Run 2, QIIME2 Run 1, and QIIME2 Run 2).

Using Mothur, sequences were denoised, filtered, and aligned to the V3-V4 region of reference bacterial sequences using the Silva 132 database (Quast et al., 2013)⁠. Chimeras were detected and removed using *chimera.vsearch* (Rognes, Flouri, Nichols, Quince, & Mahé, 2016)⁠. The pair-wise distances between each sequence were calculated at a 97% similarity and clustered into Operational Taxonomic Units (OTUs). Any sequences classified as something other than bacteria or that were unclassified at the order-level were removed from the dataset and each library was rarefied to an equal sequencing depth.

The first run resulted in a total of 5,901,931 16S rRNA gene sequences being obtained with a mean value of 75,655 ± 16,821 reads per sample (Supplementary Table 2). Of the total number of sequences, 7.7% were removed as being chimeric, resulting in an end total of 1,367,873 unique sequences. These were clustered into 853,146 single OTUs that were then rarefied to 25,130 sequences per sample.

The second run resulted in a total of 4,266,439 16S rRNA gene sequences with a mean value of 54,693 ± 11,563 reads per sample (Supplementary Table 2). Of the total number of sequences, 7.0% were removed as being chimeric, resulting in an end total of 635,439 unique sequences. These were clustered into 808,002 single OTUs that were then rarefied to 25,932 sequences per sample.

Steps of analysis can be found in the main text body for QIIME2. Between the two programs, there were noted differences in read quality filtering, clustering methods, and chimera detection. QIIME2 takes advantage of the *dada2* wrapper for filtering, dereplication, and chimera identification (Callahan et al., 2016). The raw reads are inputed and the result is a dataset ready for visualization. In Mothur, these steps are done individually, allowing the user more flexibility to understand and manipulate the formula to achieve certain results. QIIME2 clusters the data into Amplicon Sequence Variants (ASVs), which uses a *de novo* clustering method whereby biological sequences are detected by relative abundance and variants are identified by differences in a single base pair (Callahan et al., 2017). Mothur clusters by Operational Taxonomic Units (OTUs) and thus uses a *de novo* clustering method based on a dissimilarity threshold of 3% calculated by the pairwise differences between sequences (Westcott & Schloss, 2015). Chimera detection in Mothur is achieved by using *vsearch* and the Needleman–Wunsch algorithm for global alignment. Since *vsearch* was designed to identify the OTU threshold and QIIME2 clusters by ASVs, chimera detection is achieved through the *dada2* wrapper for QIIME2.

Individual-based rarefaction curves for both pipelines showed different patterns. The QIIME2 Run 1 dataset resulted in approximately 10,000 reads per sample post-rarefaction and reached a sampling plateau, whereas the Mothur Run 1 dataset resulted in approximately 25,000 reads per sample and did not reach this plateau (Supplementary Text 1, Figure 1). This pattern was observed for both Run 1 and Run 2.

Generally, both the Mothur and QIIME2 pipelines produced the same results for the muskoxen microbiome. Non-metric multidimensional scaling (NMDS) plots visualized the similarity between the two pipelines, as well as between the different sequencing runs (Supplementary Figure 1). Further, both pipelines resulted in the same dominant bacteria at the order level: *Clostridiales* were the most abundant in the bacterial community, followed by *Bacteroidales* for both pipelines. However, the relative abundance between the two leading orders varied; Mothur had an average abundance of 75% and 16% while for QIIME2 the abundance was 81% and 6%, respectively. When comparing orders with >1% abundance, QIIME2 had more bacterial orders (23 vs. 19) and both pipelines detected different combinations of orders (Supplementary Text 1, Figure 2), even though Mothur had a higher total number of orders (64 vs. 55). Most notably, Mothur detected *Actinomycetes* in Victoria Island samples post-2009, while QIIME2 detected *Microccales* and *Saccharimondales* (Supplementary Text 1, Figure 2).

After confirming the replicability of our experiment, we wanted to merge the two replicate runs for a more comprehensive, final run. This larger dataset was able to be merged and analyzed via QIIME2 and the *dada2* wrapper (9,958 total ASVs rarefied to 39,059 reads per sample), however we could not obtain enough computer power to analyze the merged dataset with Mothur. The required RAM memory to analyze this dataset surpassed our available resources (128 GB RAM), and therefore diversity indices for this dataset could only be reported using the QIIME2 pipeline. This became a limiting factor in our ability to fully compare both pipelines.

Previous studies have observed that different pipelines can provide different advantages/disadvantages when analyzing large datasets (Mysara et al., 2017; Plummer & Twin, 2015)⁠. For example, QIIME2 has been known to produce a high number of false-positives while being significantly faster than Mothur, which is more conservative and produces a higher number of unclassified reads (Mysara et al., 2017)⁠. Our study used both QIIME2 paired with the dada2 wrapper and Mothur and found that the abundances of the main bacterial phyla detected by the programs were comparable (Plummer & Twin, 2015)⁠, although only the rarefaction curve from QIIME2 reached a plauteau. QIIME2 is a user-friendly program that can quickly process large sample sizes as it detects 85x less taxonomic groups than Mothur, making it a faster and less memory-intensive option. With our computational capabilities being a limiting factor at 128 GB of usable memory, Mothur was not able to process a combined dataset.


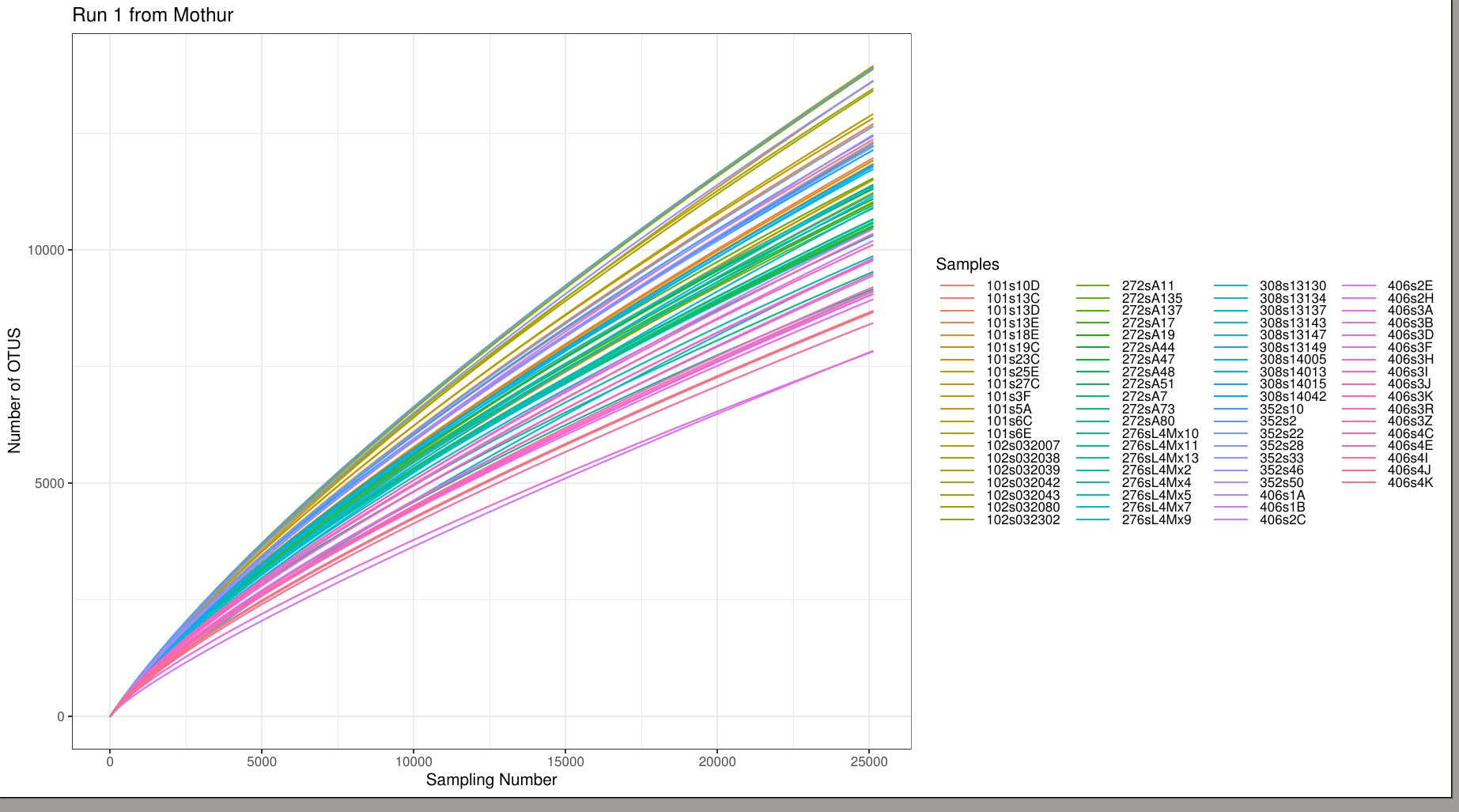

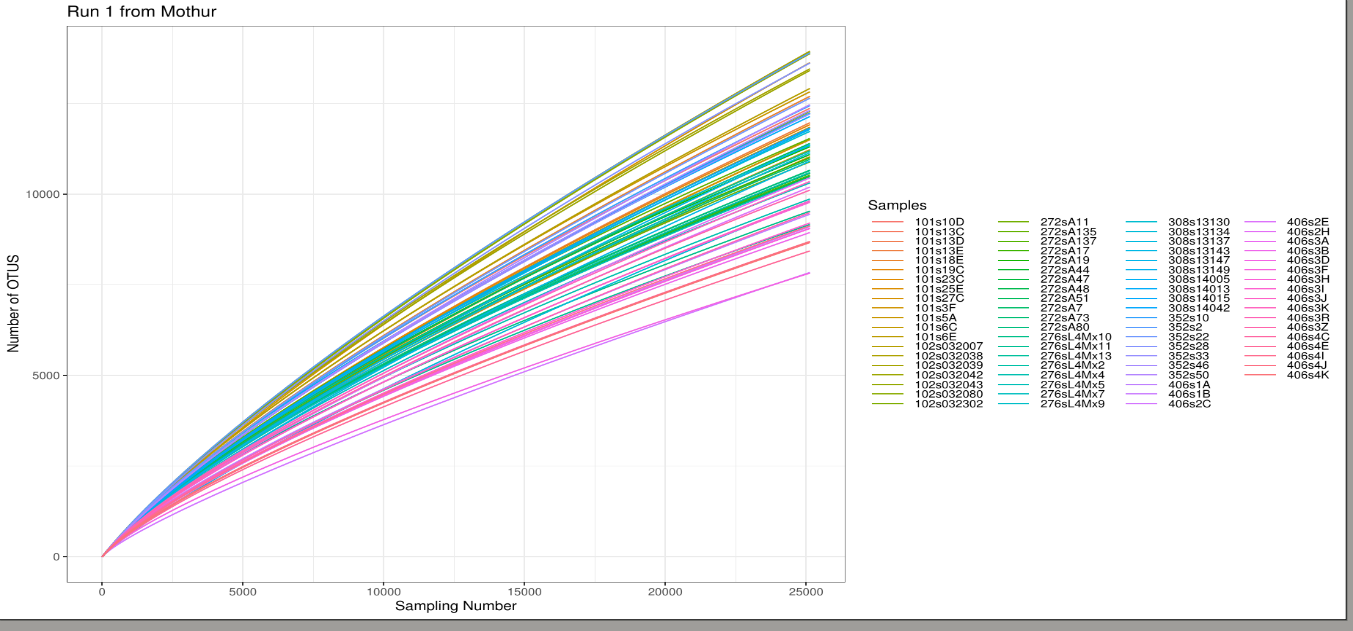

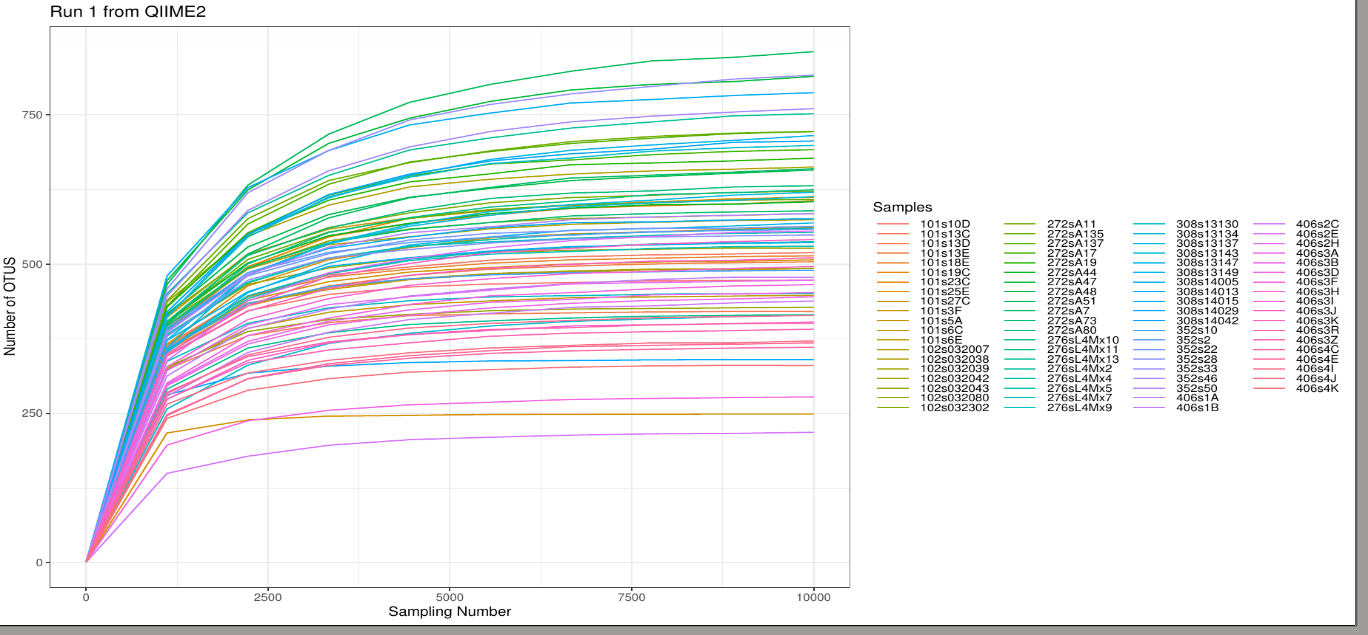


Supplementary Text Figure 1. Individual-based OTU rarefaction curves for Mothur (top) and ASV rarefaction curves for QIIME2 (bottom).


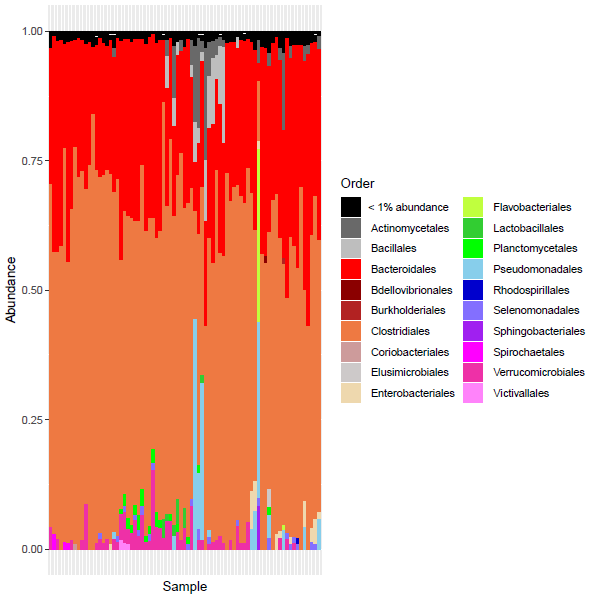


2007

2016

2009

2015

Supplementary Text Figure 2. Taxonomic relative abundance plots of the bacterial orders from the first sequencing run as classified by Mothur. Samples are organized by year of collection.
