# Supplementary material for: Geography, seasonality, and host‐associated population structure influence the fecal microbiome of a genetically depauparate Arctic mammal
Source: Ecol Evol. 2019 Nov 12;9(23):13202–17. doi: 10.1002/ece3.5768 (PMC6912892; doi:10.1002/ece3.5768)
Supplement: Supplementary file 2 [file ECE3-9-13202-s002.pdf]

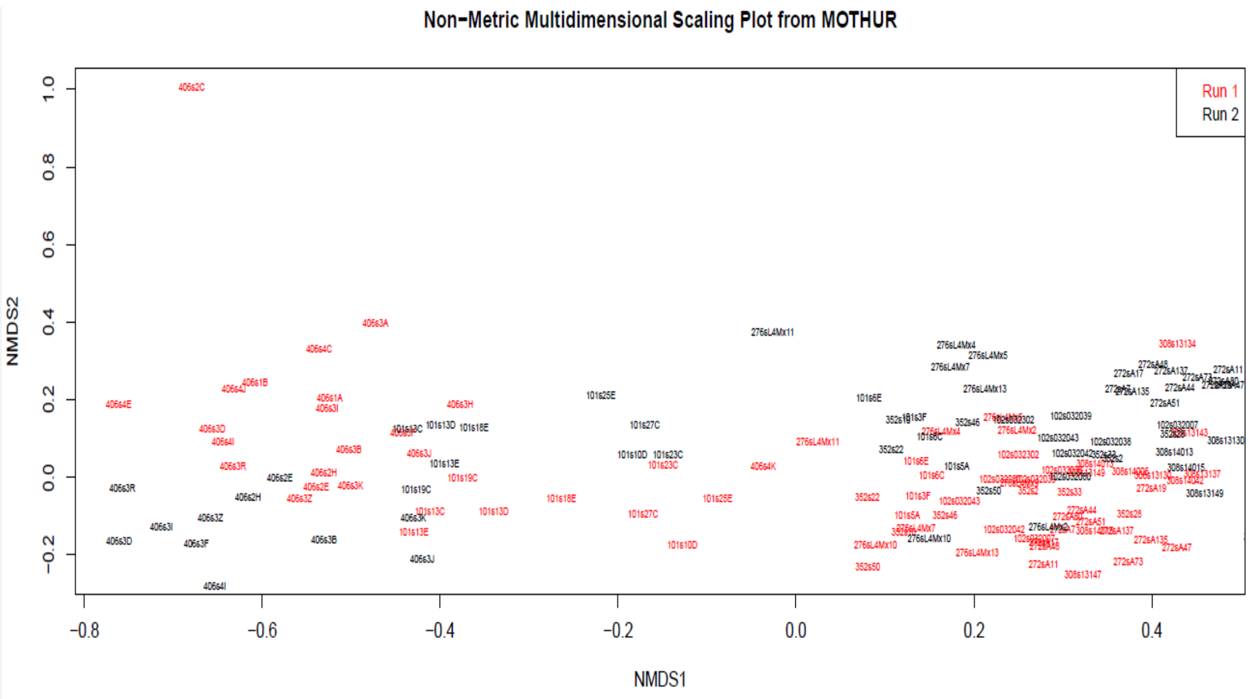

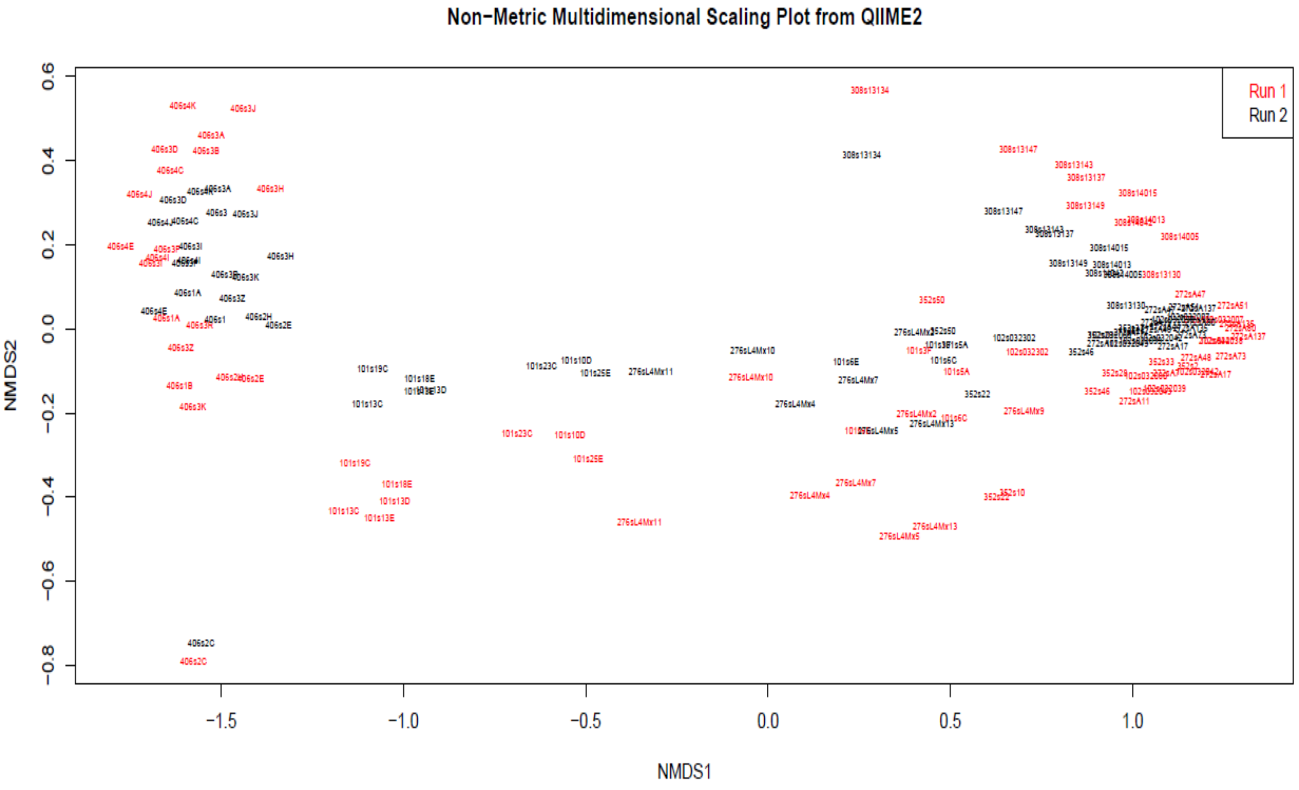

Supplementary Figure 1 – NMDS plots were used to visualize the similarity between the samples from different pipelines (above Mothur, below: QIIME2) and between runs. The stress value for QIIME2 dataset was 5.51 and the Mothur dataset resulting in a stress value of 15.35.

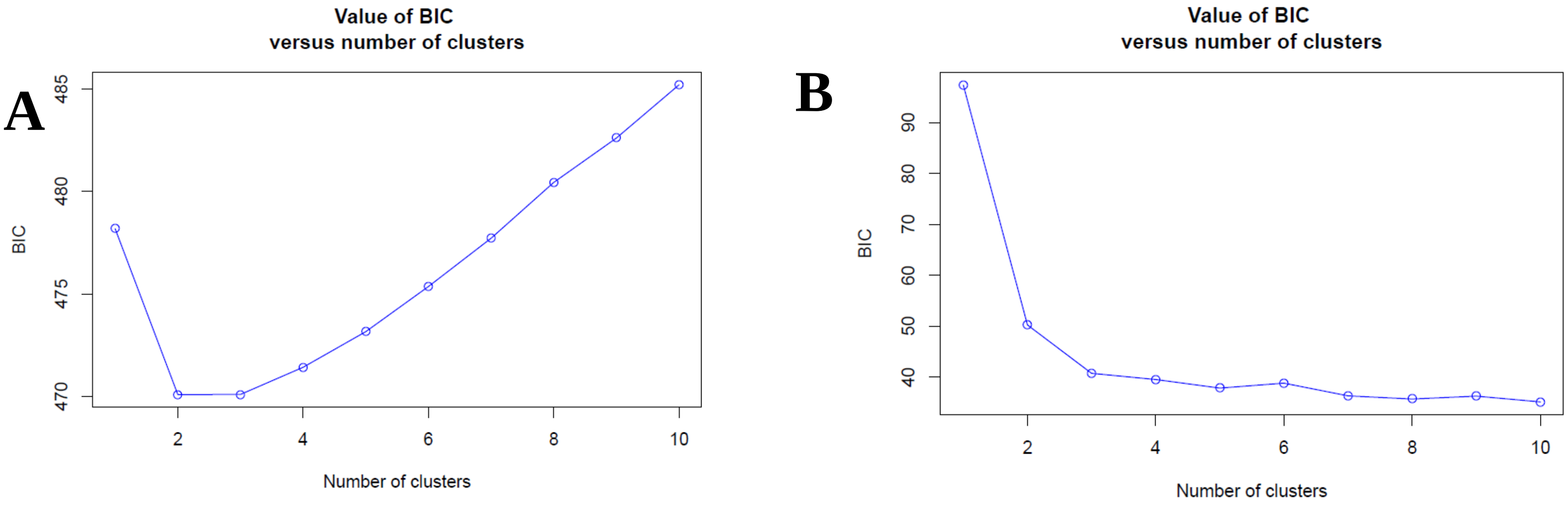

Supplementary Figure 2 – Bayesian Information Criterion (BIC) plots for various cluster numbers for microbiome (A) and microsatellite (B) data.

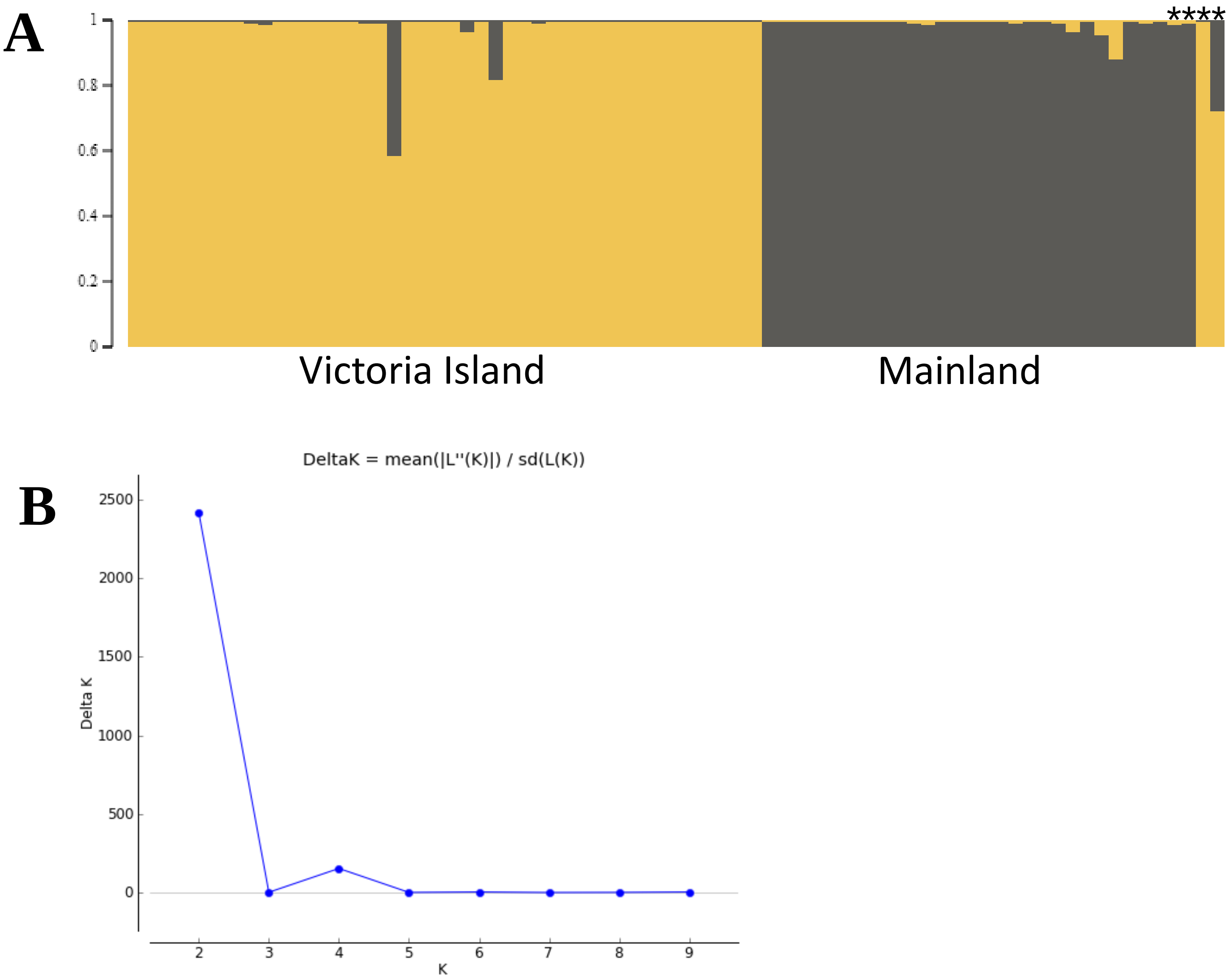

Supplementary Figure 3 – (A) STRUCTURE plot showing the number of genetic cluster (K=2) for microsatellite loci. Each column represents a different individual, and the colors represent the assigned population to each sample. Samples are grouped by population of origin (Victoria Island or Mainland). Putative migrant samples are identified by asterisks, shown above the bars. (B) Delta K plot was used to estimate the best number of clusters (K) based on microsatellite data.
